# Observational study: evaluating symptom control in allergic rhinitis at three years after starting immunotherapy

**DOI:** 10.1186/2045-7022-3-S2-P46

**Published:** 2013-07-16

**Authors:** Valerie Droessaert, Marieke Timmermans, Jan Ceuppens, Mark Jorissen, Peter Hellings

**Affiliations:** 1 University Hospitals of Leuven, Leuven, Belgium; 2 University Hospitals of Leuven, ENT Department, Leuven, Belgium; 3 University Hospitals of Leuven, Allergology Department, Leuven, Belgium

## Background

Treatment for allergic rhinitis (AR) aims at reducing the burden of allergic inflammation, either by suppression of the nasal inflammation and symptoms with pharmacotherapy or by inducing tolerance to the allergens via immunotherapy (IT). At present, we lack information on the comparison in the degree of symptom control in patients with allergic rhinitis induced by medical treatment versus immunotherapy.

## Aims

We conducted an observational study evaluating the degree of symptom control, the current medication use and the total and individual nasal symptom severity in patients at three years after either starting medical treatment or immunotherapy for AR.

## Methods

120 patients diagnosed with AR between October 2007 and February 2010 at the Ear, Nose and Throat Unit and Allergology Department of the University Hospitals of Leuven, Belgium were sent a questionnaire involving the following issues: overall satisfaction of IT, side effects caused by IT, current medication use, presence of total nasal symptoms, how much it interferes with daily activities, sleep or work and how often they still experience those symptoms. A non-IT group of 352 patients with AR treated with pharmacotherapy for 3 years was sent the same questionnaire. A visual analog scale for total nasal symptom (TNS) severity was used as a quantitative assessment of the symptoms and the proposed cut-off value of 5/10 to distinguish between controlled and uncontrolled AR was considered.

## Results

A response rate of 68% was obtained. In the IT group, based on the proposed cut-off value of VAS < 5/10 for TNS, allergic rhinitis was considered as being controlled in 69 patients (84%). Mean VAS scores in the IT group were significantly lower compared with the non-IT group. 78% of the patients treated with IT was considered as having a mild AR versus 31,5% of the non-IT patients and 81,7% an intermittent AR versus 49,4% in the non-IT group. In the IT group 69,5% of the patients didn't use any medical treatment 3 years after diagnosis of AR versus 38,9% in the non-IT group.

## Conclusions

The AR of 84% of patients three years after treatment with IT is controlled, with significantly better degree of control and less need for medical treatment compared to AR patients who received pharmacotherapy for three years. These date reinforce the need for considering treatment with IT in patients diagnosed with moderate-to-severe AR.

